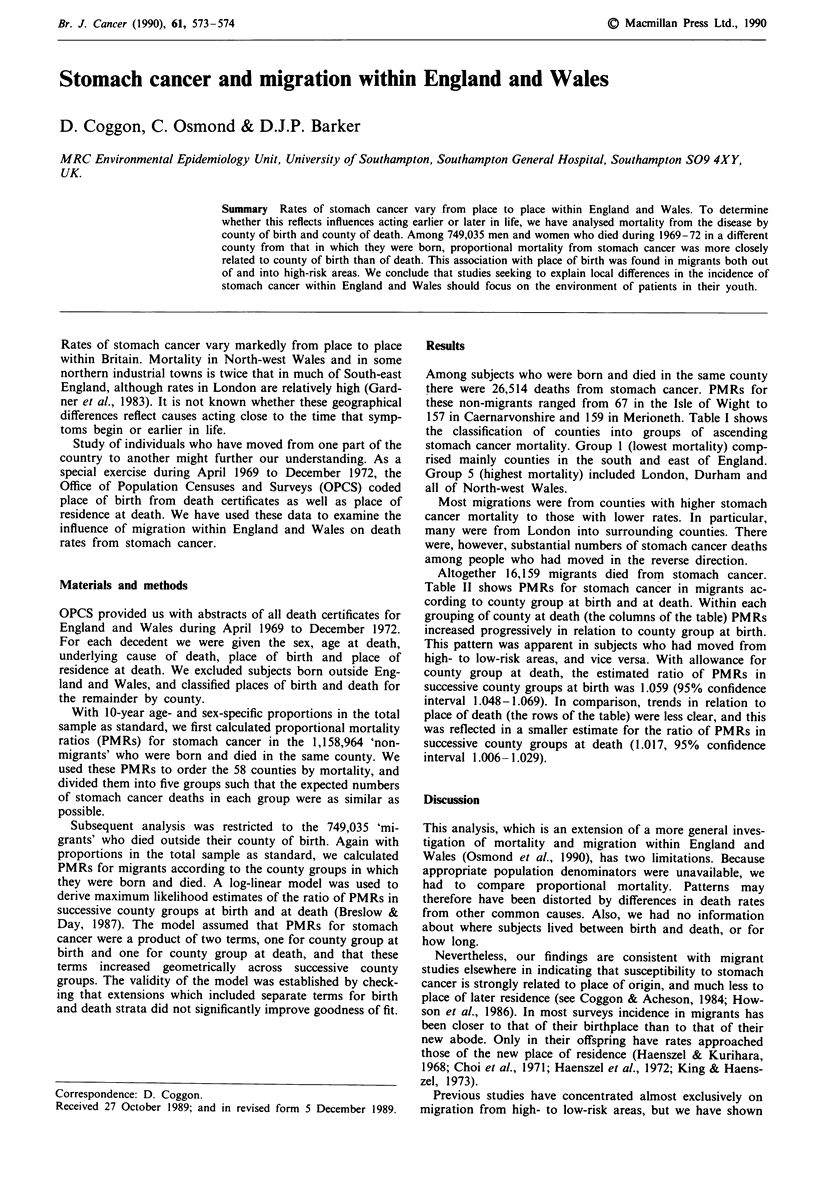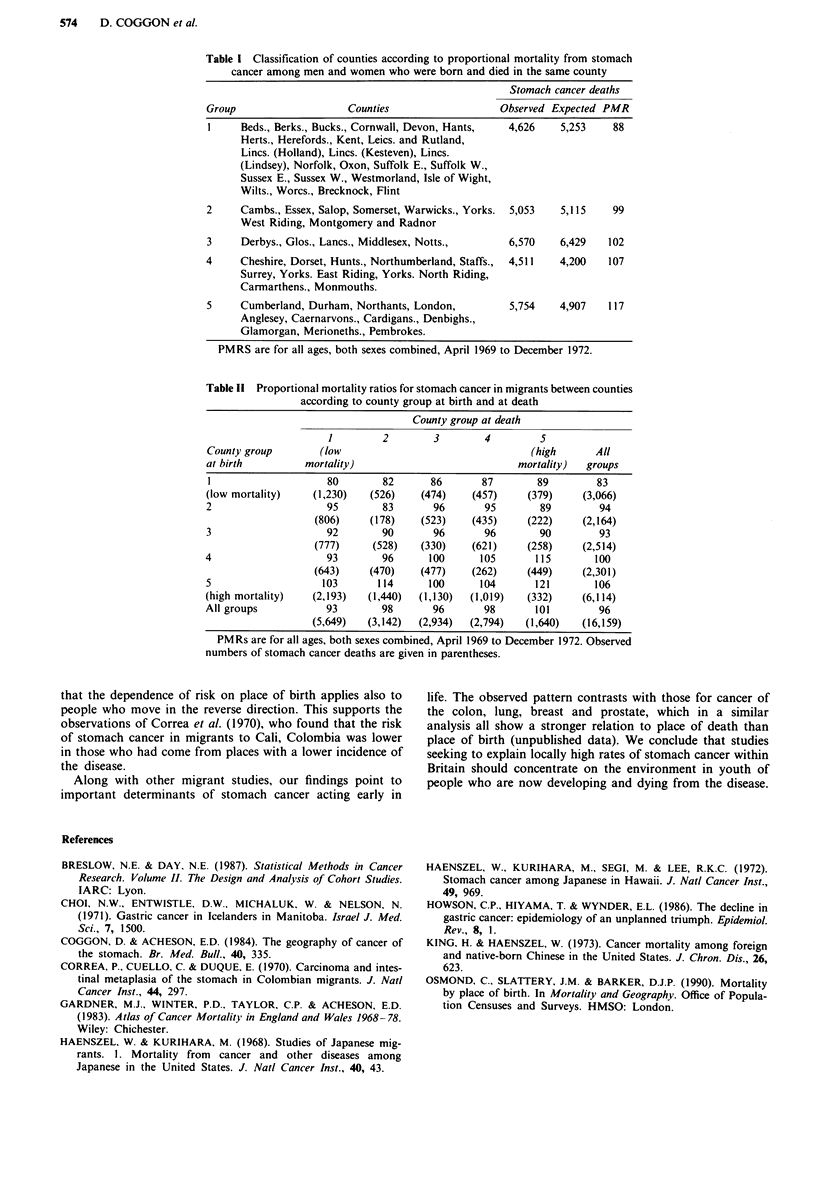# Stomach cancer and migration within England and Wales.

**DOI:** 10.1038/bjc.1990.128

**Published:** 1990-04

**Authors:** D. Coggon, C. Osmond, D. J. Barker

**Affiliations:** MRC Environmental Epidemiology Unit, University of Southampton, Southampton General Hospital, UK.

## Abstract

Rates of stomach cancer vary from place to place within England and Wales. To determine whether this reflects influences acting earlier or later in life, we have analysed mortality from the disease by county of birth and county of death. Among 749,035 men and women who died during 1969-72 in a different county from that in which they were born, proportional mortality from stomach cancer was more closely related to county of birth than of death. This association with place of birth was found in migrants both out of and into high-risk areas. We conclude that studies seeking to explain local differences in the incidence of stomach cancer within England and Wales should focus on the environment of patients in their youth.


Br. J. Cancer (1990), 61, 573–574 © Macmillan Press Ltd., 1990

### Stomach cancer and migration within England and Wales

D. Coggon, C. Osmond & D.J.P. Barker

*MRC Environmental Epidemiology Unit, University of Southampton, Southampton General Hospital, Southampton SO9 4XY, UK.*

**Summary** Rates of stomach cancer vary from place to place within England and Wales. To determine whether this reflects influences acting earlier or later in life, we have analysed mortality from the disease by county of birth and county of death. Among 749,035 men and women who died during 1969–72 in a different county from that in which they were born, proportional mortality from stomach cancer was more closely related to county of birth than of death. This association with place of birth was found in migrants both out of and into high-risk areas. We conclude that studies seeking to explain local differences in the incidence of stomach cancer within England and Wales should focus on the environment of patients in their youth.

Rates of stomach cancer vary markedly from place to place within Britain. Mortality in North-west Wales and in some northern industrial towns is twice that in much of South-east England, although rates in London are relatively high (Gardner *et al.*, 1983). It is not known whether these geographical differences reflect causes acting close to the time that symptoms begin or earlier in life.

Study of individuals who have moved from one part of the country to another might further our understanding. As a special exercise during April 1969 to December 1972, the Office of Population Censuses and Surveys (OPCS) coded place of birth from death certificates as well as place of residence at death. We have used these data to examine the influence of migration within England and Wales on death rates from stomach cancer.

### Materials and methods

OPCS provided us with abstracts of all death certificates for England and Wales during April 1969 to December 1972. For each decedent we were given the sex, age at death, underlying cause of death, place of birth and place of residence at death. We excluded subjects born outside England and Wales, and classified places of birth and death for the remainder by county.

With 10-year age- and sex-specific proportions in the total sample as standard, we first calculated proportional mortality ratios (PMRs) for stomach cancer in the 1,158,964 'non-migrants' who were born and died in the same county. We used these PMRs to order the 58 counties by mortality, and divided them into five groups such that the expected numbers of stomach cancer deaths in each group were as similar as possible.

Subsequent analysis was restricted to the 749,035 'migrants' who died outside their county of birth. Again with proportions in the total sample as standard, we calculated PMRs for migrants according to the county groups in which they were born and died. A log-linear model was used to derive maximum likelihood estimates of the ratio of PMRs in successive county groups at birth and at death (Breslow & Day, 1987). The model assumed that PMRs for stomach cancer were a product of two terms, one for county group at birth and one for county group at death, and that these terms increased geometrically across successive county groups. The validity of the model was established by checking that extensions which included separate terms for birth and death strata did not significantly improve goodness of fit.

Correspondence: D. Coggon.
Received 27 October 1989; and in revised form 5 December 1989.

### Results

Among subjects who were born and died in the same county there were 26,514 deaths from stomach cancer. PMRs for these non-migrants ranged from 67 in the Isle of Wight to 157 in Caernarvonshire and 159 in Merioneth. Table I shows the classification of counties into groups of ascending stomach cancer mortality. Group 1 (lowest mortality) comprised mainly counties in the south and east of England. Group 5 (highest mortality) included London, Durham and all of North-west Wales.

Most migrations were from counties with higher stomach cancer mortality to those with lower rates. In particular, many were from London into surrounding counties. There were, however, substantial numbers of stomach cancer deaths among people who had moved in the reverse direction.

Altogether 16,159 migrants died from stomach cancer. Table II shows PMRs for stomach cancer in migrants according to county group at birth and at death. Within each grouping of county at death (the columns of the table) PMRs increased progressively in relation to county group at birth. This pattern was apparent in subjects who had moved from high- to low-risk areas, and vice versa. With allowance for county group at death, the estimated ratio of PMRs in successive county groups at birth was 1.059 (95% confidence interval 1.048–1.069). In comparison, trends in relation to place of death (the rows of the table) were less clear, and this was reflected in a smaller estimate for the ratio of PMRs in successive county groups at death (1.017, 95% confidence interval 1.006–1.029).

### Discussion

This analysis, which is an extension of a more general investigation of mortality and migration within England and Wales (Osmond *et al.*, 1990), has two limitations. Because appropriate population denominators were unavailable, we had to compare proportional mortality. Patterns may therefore have been distorted by differences in death rates from other common causes. Also, we had no information about where subjects lived between birth and death, or for how long.

Nevertheless, our findings are consistent with migrant studies elsewhere in indicating that susceptibility to stomach cancer is strongly related to place of origin, and much less to place of later residence (see Coggon & Acheson, 1984; Howson *et al.*, 1986). In most surveys incidence in migrants has been closer to that of their birthplace than to that of their new abode. Only in their offspring have rates approached those of the new place of residence (Haenszel & Kurihara, 1968; Choi *et al.*, 1971; Haenszel *et al.*, 1972; King & Haenszel, 1973).

Previous studies have concentrated almost exclusively on migration from high- to low-risk areas, but we have shown

**Table I** Classification of counties according to proportional mortality from stomach cancer among men and women who were born and died in the same county

| Group | Counties | Stomach cancer deaths Observed | Expected | PMR |
|---|---|---|---|---|
| 1 | Beds., Berks., Bucks., Cornwall, Devon, Hants, Herts., Herefords., Kent, Leics. and Rutland, Lincs. (Holland), Lincs. (Kesteven), Lincs. (Lindsey), Norfolk, Oxon, Suffolk E., Suffolk W., Sussex E., Sussex W., Westmorland, Isle of Wight, Wilts., Worcs., Brecknock, Flint | 4,626 | 5,253 | 88 |
| 2 | Cambs., Essex, Salop, Somerset, Warwicks., Yorks. West Riding, Montgomery and Radnor | 5,053 | 5,115 | 99 |
| 3 | Derbys., Glos., Lancs., Middlesex, Notts., | 6,570 | 6,429 | 102 |
| 4 | Cheshire, Dorset, Hunts., Northumberland, Staffs., Surrey, Yorks. East Riding, Yorks. North Riding, Carmarthens., Monmouths. | 4,511 | 4,200 | 107 |
| 5 | Cumberland, Durham, Northants, London, Anglesey, Caernarvons., Cardigans., Denbighs., Glamorgan, Merioneths., Pembrokes. | 5,754 | 4,907 | 117 |

PMRS are for all ages, both sexes combined, April 1969 to December 1972.

**Table II** Proportional mortality ratios for stomach cancer in migrants between counties according to county group at birth and at death

| County group at birth | County group at death 1 (low mortality) | 2 | 3 | 4 | 5 (high mortality) | All groups |
|---|---|---|---|---|---|---|
| 1 | 80 | 82 | 86 | 87 | 89 | 83 |
| (low mortality) | (1,230) | (526) | (474) | (457) | (379) | (3,066) |
| 2 | 95 | 83 | 96 | 95 | 89 | 94 |
| | (806) | (178) | (523) | (435) | (222) | (2,164) |
| 3 | 92 | 90 | 96 | 96 | 90 | 93 |
| | (777) | (528) | (330) | (621) | (258) | (2,514) |
| 4 | 93 | 96 | 100 | 105 | 115 | 100 |
| | (643) | (470) | (477) | (262) | (449) | (2,301) |
| 5 | 103 | 114 | 100 | 104 | 121 | 106 |
| (high mortality) | (2,193) | (1,440) | (1,130) | (1,019) | (332) | (6,114) |
| All groups | 93 | 98 | 96 | 98 | 101 | 96 |
| | (5,649) | (3,142) | (2,934) | (2,794) | (1,640) | (16,159) |

PMRs are for all ages, both sexes combined, April 1969 to December 1972. Observed numbers of stomach cancer deaths are given in parentheses.

that the dependence of risk on place of birth applies also to people who move in the reverse direction. This supports the observations of Correa *et al.* (1970), who found that the risk of stomach cancer in migrants to Cali, Colombia was lower in those who had come from places with a lower incidence of the disease.

Along with other migrant studies, our findings point to important determinants of stomach cancer acting early in life. The observed pattern contrasts with those for cancer of the colon, lung, breast and prostate, which in a similar analysis all show a stronger relation to place of death than place of birth (unpublished data). We conclude that studies seeking to explain locally high rates of stomach cancer within Britain should concentrate on the environment in youth of people who are now developing and dying from the disease.

### References

BRESLOW, N.E. & DAY, N.E. (1987). *Statistical Methods in Cancer Research. Volume II. The Design and Analysis of Cohort Studies.* IARC: Lyon.

CHOI, N.W., ENTWISTLE, D.W., MICHALUK, W. & NELSON, N. (1971). Gastric cancer in Icelanders in Manitoba. *Israel J. Med. Sci.*, **7**, 1500.

COGGON, D. & ACHESON, E.D. (1984). The geography of cancer of the stomach. *Br. Med. Bull.*, **40**, 335.

CORREA, P., CUELLO, C. & DUQUE, E. (1970). Carcinoma and intestinal metaplasia of the stomach in Colombian migrants. *J. Natl Cancer Inst.*, **44**, 297.

GARDNER, M.J., WINTER, P.D., TAYLOR, C.P. & ACHESON, E.D. (1983). *Atlas of Cancer Mortality in England and Wales 1968–78.* Wiley: Chichester.

HAENSZEL, W. & KURIHARA, M. (1968). Studies of Japanese migrants. 1. Mortality from cancer and other diseases among Japanese in the United States. *J. Natl Cancer Inst.*, **40**, 43.

HAENSZEL, W., KURIHARA, M., SEGI, M. & LEE, R.K.C. (1972). Stomach cancer among Japanese in Hawaii. *J. Natl Cancer Inst.*, **49**, 969.

HOWSON, C.P., HIYAMA, T. & WYNDER, E.L. (1986). The decline in gastric cancer: epidemiology of an unplanned triumph. *Epidemiol. Rev.*, **8**, 1.

KING, H. & HAENSZEL, W. (1973). Cancer mortality among foreign and native-born Chinese in the United States. *J. Chron. Dis.*, **26**, 623.

OSMOND, C., SLATTERY, J.M. & BARKER, D.J.P. (1990). Mortality by place of birth. In *Mortality and Geography.* Office of Population Censuses and Surveys. HMSO: London.